# Millet seed oil activates β–catenin signaling and promotes hair growth

**DOI:** 10.3389/fphar.2023.1172084

**Published:** 2023-05-09

**Authors:** Eunyoung Lee, Hyo-Deok Seo, Daedong Kim, So-Hyun Park, Soo Ro Kim, Changhun Hyun, Jeong-Hoon Hahm, Tae-Youl Ha, Jiyun Ahn, Chang Hwa Jung

**Affiliations:** ^1^ Aging and Metabolism Research Group, Korea Food Research Institute, Wanju-Gun, Republic of Korea; ^2^ Department of Food Biotechnology, University of Science and Technology, Wanju-Gun, Republic of Korea; ^3^ Suheung Research Center, Seongnam-si, Republic of Korea; ^4^ Balanceway, Seoul, Republic of Korea

**Keywords:** alopecia, beta-catenin, dermal papilla cells, hair growth, millet seed oil

## Abstract

Alopecia, regardless of gender, exacerbates psychological stress in those affected. The rising prevalence of alopecia has fueled a research interest in preventing hair loss. This study investigates the potential of millet seed oil (MSO) in promoting the proliferation of hair follicle dermal papilla cells (HFDPC) and stimulating hair growth in animals with testosterone-dependent hair growth inhibition as part of a study on dietary treatments to improve hair growth. MSO-treated HFDPC significantly increased cell proliferation and phosphorylation of AKT, S6K1, and GSK3β proteins. This induces β-catenin, a downstream transcription factor, to translocate to the nucleus and increase the expression of factors related to cell growth. In a C57BL/6 mice model in which hair growth was inhibited by subcutaneous testosterone injection after shaving the dorsal skin, oral administration of MSO stimulated hair growth in the subject mice by increasing the size and number of hair follicles. These results suggest that MSO is a potent agent that may help prevent or treat androgenetic alopecia by promoting hair growth.

## 1 Introduction

In modern society, appearance is a means of expressing confidence; hair plays an important role superficially as the first part of the body seen by others. The prevalence of androgenetic alopecia varies depending on region and race; however, it is known to increase with age and affects nearly 30%–50% of men by the age 50 (Hamilton, 1951; Norwood, 2001; Cranwell and, 2015). Hair loss (alopecia) has significant aesthetic and psychological impacts. Several studies have shown that the quality of life of individuals with alopecia may be considerably hampered (Sehgal et al., 2007). Therefore, there is a growing interest in developing an effective hair loss treatment.

The hair growth cycle involves the anagen, catagen, and telogen phases. It is affected by various factors, such as excessive androgens, stress, vascular disorders, genetic factors, and aging (Alonso and Fuchs, 2006). In androgenetic alopecia, androgens are thought to shorten the anagen phase, increase the catagen and telogen phases, and delay the shift from the telogen to the anagen phase (Cotsarelis and Milar, 2001). Thus, alopecia is caused by hair cycle and follicle morphology disorders, which can lead to hair loss. Currently, minoxidil and finasteride are two representative drugs approved by the US Food and Drug Administration (FDA) for the treatment of alopecia. Minoxidil was originally developed as a vasodilator to treat hypertension; however, it is used as a hair growth promoter because hypertrichosis has been reported as a side effect (Suchonwanit et al., 2019). Later, it was formulated as a topical solution and used as a hair loss treatment. The exact mechanism of action of minoxidil has not yet been elucidated. Long-term use of minoxidil can result in various side effects, including scalp irritation, itching, dryness, and excessive facial, and body hair growth (Rossi et al., 2012). Furthermore, minoxidil has been reported to affect blood pressure and heart rate (Suchonwanit et al., 2019). Another approved drug, finasteride, is a representative 5α-reductase inhibitor that lowers blood DHT levels to slow down the progress of hair loss and promote hair growth (Kaufman and Dawber, 1999). Testosterone, known as one of the causes of hair loss, forms dihydrotestosterone (DHT) by the action of 5α-reductase, which negatively affects hair follicles (Chen et al., 2020). Effective hair loss treatment requires continuous administration, and there are still concerns about the potential long-term side effects of finasteride, such as sexual dysfunction (Irwig and Kolukula, 2011). Therefore, there is a need for a potent alternative drug that is safe and effective. Thus, studies using materials with fewer side effects that help hair health without the need for a prescription for hair loss, which requires long-term treatment, are attracting attention.

The millet (Panicum miliaceum) seed is one of the first cultivated plants; today, they are grown primarily in Asia, European countries, and Russia, and it is attracting attention as a new substitute crop and food ingredient. They contain more essential amino acids than barley, oats, or wheat (Kalinová, 2007). Additionally, millet has various health benefits, which include the following. (1) Millet seed is a source of antioxidants and anti-inflammatory agents that protect the body from free radicals and inflammatory damage to cells (Chandrasekara and Shahidi, 2012). (2) Some studies have suggested that millet seed has anticancer properties, particularly in breast and colon cancers (Zhang et al., 2014; Shan et al., 2015). (3) Millet seed has a low glycemic index, which can help to control blood sugar levels in people with diabetes (Bangar et al., 2021). (4) Millet seed is naturally gluten-free and can be an excellent alternative to wheat-based products for those with gluten sensitivity or celiac disease (Bielecka et al., 2022). (5) Millet seed is a good source of micronutrients, such as iron, zinc, and magnesium (Govindaraj et al., 2022). In addition, millet seed is a nutritious food commonly consumed daily and has a higher oil content (4%–9%) than all other grains (Jain and Bal, 1997). Millet seed oil (MSO) contains linoleic acid, vitamin E, and phytosterols. Miliacin, a representative phytosterol of MSO, has been reported to promote keratinocyte proliferation (Obrigkeit et al., 2006). In clinical trials, miliacin supplementation significantly reduced the telogen phase and improved scalp dryness and hair conditions (Keophiphath et al., 2020). These properties of miliacin suggest that MSO might have potential benefits for maintaining healthy hair by contributing to hair growth. However, the effect and mechanism of MSO on healthy hair growth have not been elucidated and further research was needed on this.

The β-catenin signaling pathway is the primary therapeutic target for hair loss treatment. Through cell growth and differentiation, β-catenin promotes the anagen phase and hair growth in papilla cells (Choi, 2020). β-Catenin is regulated by various factors, such as axis inhibition protein (Axin)/adenomatous polyposis coli (APC), glycogen synthase kinase 3 beta (GSK3β) and Wnt ligands (Van Der Lugt et al., 2019). β-Catenin is controlled by its ubiquitination and proteasomal degradation. Axin/APC and GSK3β promote β-catenin degradation (Sun et al., 2001). β-catenin phosphorylated by the Axin/APC/GSK3β complex is recognized by the E3 ubiquitin ligase TrCP1 (also called β-TrCP) and degraded by the proteasome to regulate the amount of β-catenin in cells. (Wu et al., 2009). The Wnt ligand is a representative activator of β-catenin. When Wnt signaling is activated, it phosphorylates GSK3β to inhibit its activity, increasing the β-catenin levels and causing its accumulation in the nucleus, where it functions as a transcriptional cofactor to promote the expression of cell growth factors by binding to Tcf/Lef transcription factors (Brock, 2014). The mechanistic target of rapamycin complex 1 (mTORC1) is a serine/threonine protein kinase that integrates numerous environmental signals. The most common function of mTORC1 is to promote cell proliferation and growth (Laplante and Sabatini, 2012). Studies have shown that mTORC1 plays a key role in regulating the proliferation and differentiation of dermal cells, and its activity increases during the anagen phase of the hair follicle cycle (Deng et al., 2015; Wang et al., 2022). These results suggest that mTORC1 may play an essential role in promoting the growth and development of new hair by stimulating the proliferation and differentiation of dermal cells.

In this study, we investigated the potential of MSO in promoting cell proliferation in human hair follicle dermal papilla cells (HFDPCs) and increasing hair growth in the shaved-back skin of C57BL/6 mice. Our study also aimed to examine the effects of MSO on hair growth through its involvement in β-catenin signaling, and test its potential as a viable treatment or preventative measure for alopecia.

## 2 Materials and methods

### 2.1 MSO

Millet (*P. miliaceum* L.) seeds were dried and powdered. The millet seed powder was then subjected to supercritical extraction using CO_2_ to produce MSO (Devittori et al., 2000). MSO comprises various fatty acids such as palmitic acid C16:0, palmitoleic acid C16:1 w7, stearic acid C18:0, oleic acid C18:1 w9, vaccenic acid C18:1 w7, linoleic acid C18:2 w6, alpha-linolenic acid C18:3 w3, and arachidic acid C20:0 (Supplementary Material).

### 2.2 Cell culture

HFDPCs purchased from Cefobio (Seoul, Korea) were grown in Dulbecco’s Modified Eagle Medium (DMEM) with 10% fetal bovine serum and 1% penicillin/streptomycin at 37 C in a 5% CO_2_ incubator.

### 2.3 Cell proliferation

To investigate MSO effect on the cell proliferation, MTT and BrdU assays were conducted. MTT assay was used at passage 4 of HFDPCs. HFDPCs were seeded into 96-well plates at a cell density of 1 × 10^4^ cells/well. The cells were treated with MSO at concentrations of 0, 12.5, 25, 50, 100, and 200 μg/mL for 24 h. Wells without MSO acted as the control. The stock solution of MTT was prepared with 5 mg/mL in phosphate buffered saline (PBS), filtered, and added to the medium at a ratio of 1:10, and allowed to react for 2 h. The wavelength was measured at 570 nm. The BrdU cell proliferation assay were performed according to the manufacturer’s instructions (Sigma-Aldrich, MO, United States). Briefly, the BrdU assay kit measures 5-bromo-2′-deoxyuridine (BrdU) incorporated into the cellular DNA during cell growth. When cells were maintained in DMEM containing BrdU, this pyrimidine analog would be incorporated into the DNA of proliferating cells instead of thymidine. The absorbance was measured using an ELISA reader at 450 nm single wavelength, and the magnitude of the absorbance was related to the amount of BrdU incorporated into the cells. In addition, mTORC1 activity is tested in nutrition-starved condition. HFDPCs were seeded into 6-well plates at a cell density of 1.5 × 10^5^ cells/well. After incubating for 24 h, the medium was changed to FBS-free medium and incubated for 2 h. MSO was treated at a concentration of 25 μg/mL at time intervals to observe changes in markers in a state in which nutrients were depleted.

### 2.4 Animal experiments

Six-week-old male C57BL/6 mice (*n* = 40) were purchased from Orient Bio, Inc. (Seongnam, Korea) and provided with a chow diet with access to fresh water. The mice were maintained free of specific pathogens at a controlled temperature (23°C ± 2°C), humidity (50% ± 20%), and light (12 h light-dark cycle). The Institutional Animal Care and Use Committee (KFRI-M-21060) of the Korea Food Research Institute approved the animal studies. Forty mice were separated into four groups (*n* = 10/group) and left for 1 week of adaptation. After shaving the skin surface of the back of the mice, testosterone (T) was subcutaneously injected (0.5 mg per day) five times a week; then, each vehicle and MSO were orally administered five times a week. The control group was orally administered 100 μL of vehicle PBS (T + Veh) daily. The positive control group was administered finasteride, which is approved by the FDA as a hair loss treatment agent (T + Fina). Finasteride was orally administered at 0.5 mg/kg mice/day, considering the recommended dosage. MSO (100 and 200 mg/kg mice/day) was orally administered to the low- and high-dose MSO groups (T + MSO 100 and MSO 200). The degree of hair growth was observed by photographing the hair of the experimental animals once a week. After 5 weeks, the animals were sacrificed post anesthesia with a mixture of oxygen and isoflurane gas in an induction chamber. Isoflurane was used at a concentration of 4% for the induction of anesthesia and 2.0% for the maintenance of anesthesia (Flecknell et al., 2015). Hair length was measured in mm using a caliper. The liver tissue weights were measured after sacrificing the mice, and mouse serum and skin tissue were obtained. Mouse skin tissue was obtained via meticulous dissection to separate it from subcutaneous fat to minimize analysis errors. The tissues were stored in a deep freezer at −70°C until analysis.

### 2.5 mRNA analysis

Translocation of β-catenin into the nucleus results in the upregulation of transcriptional markers related to cell cycle and growth factors (Brock, 2014). Specific markers were identified through mRNA analysis, and the primer sequences of genes used for this analysis are listed in Table 1. Total RNA from HFDPCs and skin tissues was extracted using RNeasy Mini Kit (Qiagen, Valencia, CA, United States). All the experimental steps were performed according to the manufacturer’s instructions. After RNA extraction, cDNA was synthesized using the ReverTra Ace^®^ qPCR RT kit (Toyobo, Osaka, Japan), and quantitative RT-qPCR was performed using SYBR Green real-time PCR Master Mix (Toyobo, Japan). After pre-denaturation at 95°C for 1 min, denaturation (95°C for 15 s), annealing (58°C for 15 s), and extension (72°C for 45 s) were repeated for 40 cycles (Lee et al., 2021). The gene of interest was normalized to the housekeeping gene and quantified using the 2^-△△Ct^ method.

**TABLE 1:** Sequences of primers used for the quantitative reverse transcription polymerase chain reaction in this study (RT-qPCR).

Gene		Primer	Sequence (5′to 3′)
Human	*β-actin*	Forward	ACG​TCG​ACA​TCC​GCA​AAG​ACC​TC
		Reverse	TGA​TCT​CCT​TCT​GCA​TCC​GGT​CA
	*Igf-1*	Forward	TGT​CCT​CCT​CGC​ATC​TCT​TCT​ACC
		Reverse	TAA​AAG​CCC​CTG​TCT​CCA​CAC​ACG
	*Fgf-7*	Forward	TTG​TGG​CAA​TCA​AAG​GGG​TG
		Reverse	CCT​CCG​TTG​TGT​GTC​CAT​TTA​GC
	*Fgf-10*	Forward	GCA​TGT​GCG​GAG​CTA​CAA​TCA
		Reverse	ACG​GCA​ACA​ACT​CCG​ATT​TCT​AC
	*Kgf*	Forward	ATC​AGG​ACA​GTG​GCA​GTT​GGA
		Reverse	AAC​ATT​TCC​CCT​CCG​TTG​TGT
	*Vegf*	Forward	CCC​ACT​GAG​GAG​TCC​AAC​AT
		Reverse	AAA​TGC​TTT​CTC​CGC​TCT​GA
	*Dkk-1*	Forward	TTC​CGA​GGA​GAA​ATT​GAG​GA
		Reverse	CCT​GAG​GCA​CAG​TCT​GAT​GA
	*Bcl-2*	Forward	CAG​CTG​CAC​CTG​ACG​CCC​TT
		Reverse	GCC​TCC​GTT​ATC​CTG​GAT​CC
	*Bax*	Forward	ACC​AAG​AAG​CTG​AGC​GAG​TGT​C
		Reverse	TGT​CCA​GCC​CAT​GAT​GGT​TC
Mouse	*Gapdh*	Forward	TGG​ATT​TGG​ACG​CAT​TGG​TC
		Reverse	TTT​GCA​CTG​GTA​CGT​GTT​GAT
	*Igf-1*	Forward	TCA​ACA​AGC​CCA​CAG​GGT​AT
		Reverse	ACT​CGT​GCA​GAG​CAA​AGG​AT
	*Vegf*	Forward	TCT​TCA​AGC​CAT​CCT​GTG​TG
		Reverse	GCG​AGT​CTG​TGT​TTT​TGC​AG
	*Fgf-7*	Forward	AGACTGTTCTGTCGCACC
		Reverse	CCG​CTG​TGT​GTC​CAT​TTA​G
	*Fgf-10*	Forward	TGT​CCG​CTG​GAG​AAG​GCT​GTT​C
		Reverse	CTA​TGT​TTG​GAT​CGT​CAT​GG
	*Fgf-21*	Forward	CTA​TGT​TTG​GAT​CGT​CAT​GG
		Reverse	CGGCCCTGTAAAGGCTCT

Abbreviations: Insulin like growth factor 1 (*Igf-1*), fibroblast growth factor 7 (*Fgf-7*), fibroblast growth factor 10 (*Fgf-10*), fibroblast growth factor 21 (*Fgf-21*), keratinocyte growth factor (*Kgf*), vascular endothelial growth factor (*Vegf*), CCAAT-enhancer-binding protein *a* (*Dkk-1*), B cell lymphoma protein 2 (*Bcl-2*), Bcl-2, associated X protein (*Bax*).

### 2.6 Western blot analysis

Proteins were isolated from HFDPCs and skin tissue of experimental animals in the radioimmunoprecipitation assay (RIPA) lysis buffer (Thermo Fisher Scientific Pierce, Rockford, IL, United States) containing protease and phosphatase inhibitors. The protein concentration was quantified using the bicinchoninic acid (BCA) assay. Equal concentrations of protein loaded on the gel were separated by using sodium dodecyl sulfate-polyacrylamide gel electrophoresis. The separated proteins in the gel were transferred to a polyvinylidene fluoride membrane. After transfer, the membrane was incubated with 5% skimmed milk for 1 h and washed. After washing, the membrane was incubated with primary antibodies overnight at 4°C followed by incubation with secondary antibodies for 1 h. Western blot detection was performed using a chemiluminescence reagent. Antibodies against β-actin, Vinculin, Cyclin D1 (sc-8396) and Cyclin E (sc-247) were purchased from Santa Cruz Biotechnology (CA, United States). Antibodies against p-S6K1 (Thr389) (9205 s), S6K1 (2708 s), p-Akt (Ser473) (4060 s), Akt (9272 s), p-ERK (Thr202/Tyr204) (4377), ERK (4695), β-catenin (9582 s), CDK2 (2546), Cdc2 (9112 s), p-GSK-3β (Ser9) (9336 s), and GSK-3β (9315 s) were purchased from Cell Signaling Technology (Danvers, MA, United States).

### 2.7 Immunofluorescence analysis

HFDPCs were seeded into 8-well chamber slides (Thermo Fisher Scientific Pierce, Rockford, IL, United States). MSO was used at concentrations of 0, 12.5, and 25 μg/mL for 24 h. Minoxidil, known to promote the translocation of β-catenin to the nucleus in HFDPCs, was used as a positive control (Kwack et al., 2011). Briefly, the cells were fixed with 3.7% formaldehyde for 15 min and then incubated with 0.05% saponin for 30 min at 20°C–25°C to increase cell permeability. After blocking with 1% bovine serum albumin for 1 h, the cells were incubated with the primary antibody overnight at 4°C. The primary antibody was β-catenin (9582 s) purchased from Cell Signaling Technology (Danvers, MA, United States). After removing the primary antibody solution and washing, the cells were incubated with secondary antibody for 1 h at 20°C–25°C. The cells were observed under an FV3000 confocal laser scanning microscope (Olympus, Tokyo, Japan).

### 2.8 Histological analysis

The mouse skin tissues were fixed in 3.7% formaldehyde for 24 h. To the tissues were filled with paraffin to embed the samples, and the embedded tissue samples were cut into 5-µm thick sections. Tissues were then stained with hematoxylin and eosin (H&E) to easily distinguish the different tissue structures. Their morphology was evaluated using an Olympus microscope (Olympus, Tokyo, Japan) at a magnification of ×200.

### 2.9 Statistical analysis

Comparisons between groups were performed using one-way analysis of variance (ANOVA) with Tukey test for multiple comparison analysis statistics. The normality and homogeneity of variance assumptions was verified before performing the ANOVA test. The results of these tests indicated that the data met the assumptions of normality and homogeneity of variance, justifying the use of the ANOVA test. Statistical analysis was performed using GraphPad Prism Version 7.0 Software (San Diego, CA, United States), and a *p*-value <0.05 was considered statistically significant. Data are expressed as the mean ± standard error of the mean (SEM) or standard deviation.

## 3 Results

### 3.1 MSO promotes HFDPCs proliferation

**FIGURE 1:**
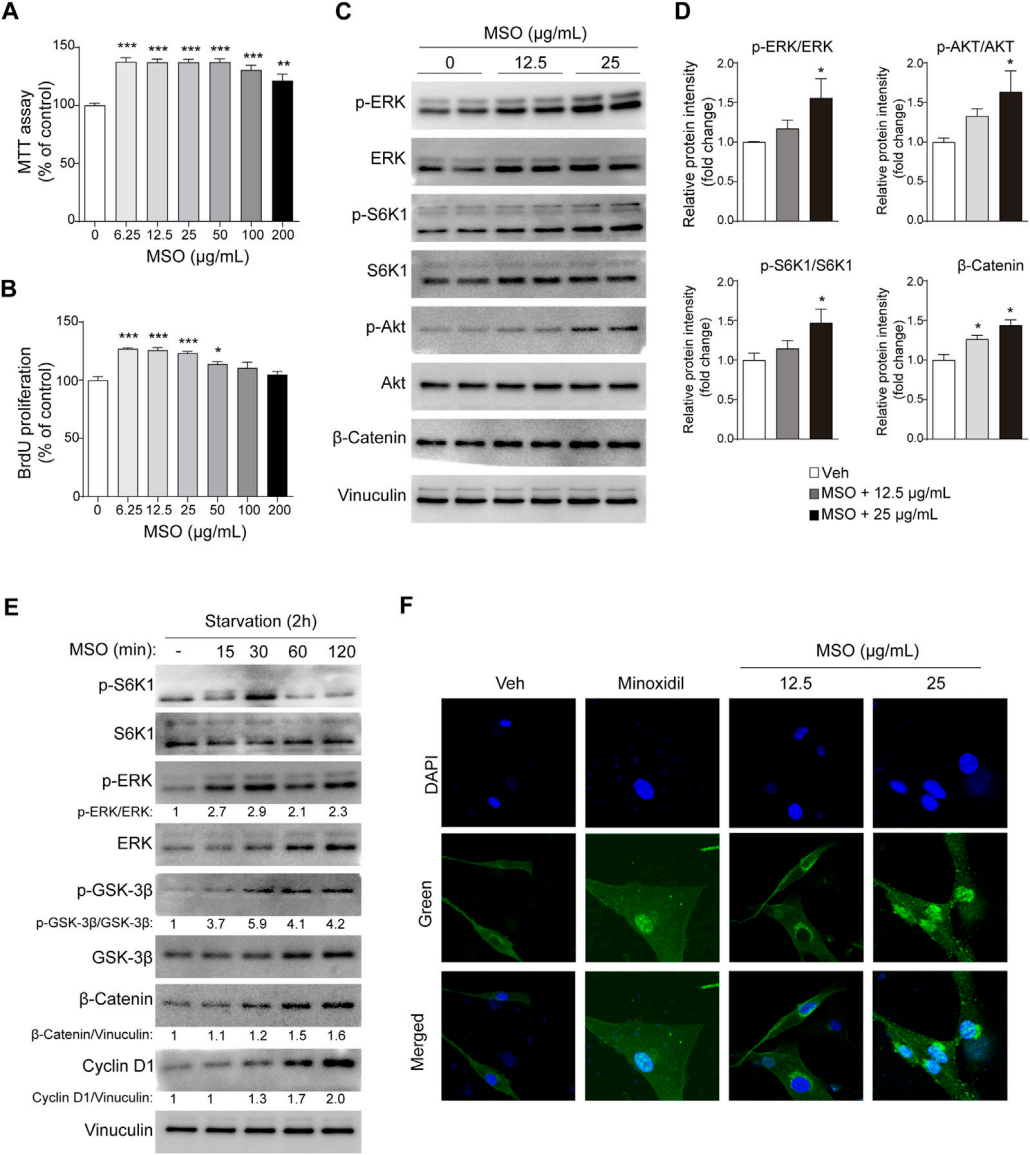
Effect of MSO on the proliferation of HFDPCs. HFDPCs were treated with MSO for 24 h. **(A)** Cell proliferation was measured using the MTT assay **(B)** BrdU cell proliferation assay evaluated cell populations that synthesize DNA. **(C)** HFDPCs were treated with MSO at concentrations of 0, 12.5, and 25 μg/mL for 24 h. Cell proliferation-related markers were analyzed by using Western blotting. **(D)** Relative protein levels of proliferation-related biomarkers were quantified using ImageJ. Values are expressed as the ratio of phospho-form protein to total-form protein and represented as mean ± SEM. **(E)** After seeding the cells and incubating for 24 h, the medium was changed to FBS-free medium. MSO was treated at a concentration of 25 μg/mL at time intervals to observe changes in markers in a state in which nutrients were depleted. The Wnt/β-catenin pathway of HFDPCs in starvation conditions was analyzed by using Western blotting. **(F)** Immunostaining was performed to evaluate the translocation of β-catenin to the nucleus in HFDPCs. *, *p* < 0.05; **, *p* < 0.01; ***, *p* < 0.001 compared to the negative control group. Abbreviations: HFDPCs, human hair follicle dermal papilla cells; CDK2, cyclin-dependent kinase 2; Cdc2, cell division cycle protein 2; ERK, extracellular signal-regulated kinase; MSO, millet seed oil; S6K1, ribosomal protein S6 kinase beta-1.

The potential of MSO to promote the growth of HFDPCs was evaluated using the MTT assay. Treatment with MSO at different concentrations (0–200 μg/mL) significantly increased the proliferation of HFDPCs at all tested concentrations (Figure 1A). BrdU cell proliferation assay suggested that the DNA synthesis of HFDPCs was also significantly increased by MSO treatment at a concentration of 6.25–50 μg/mL, compared to the control group (*p* < 0.05–0.001) (Figure 1B).

### 3.2 MSO stimulates the β-catenin and mTORC1 signaling

When MSO was used for treatment at a concentration of 25 μg, the phosphorylation of ERK, S6K1, and AKT, proteins related to cell proliferation, was significantly increased (*p* < 0.05) (Figures 1C,D). MSO treatment also increased the expression of β-catenin, which promotes the cell-cycle (*p* < 0.05) (Figures 1C,D). In addition to changes in nutrient-rich conditions, we also observed changes in these markers in HFDPCs when nutrients were limited. Phosphorylation of protein markers related to hair growth was proved when MSO was treated under conditions of metabolic stress, such as nutrient deficiency. We confirmed that the protein expression of cell proliferation-related markers, including mTORC1 signaling, increased when MSO was administered for each hour in a state in which nutrients were depleted (Figure 1E). When observed under a fluorescence microscope, it was confirmed that β-catenin translocates into the cell nucleus following MSO treatment (Figure 1F). Collectively, we show that MSO significantly increased Wnt/β-catenin and mTORC1 signaling.

### 3.3 MSO activates cell cycle progression and induces growth factors

**FIGURE 2:**
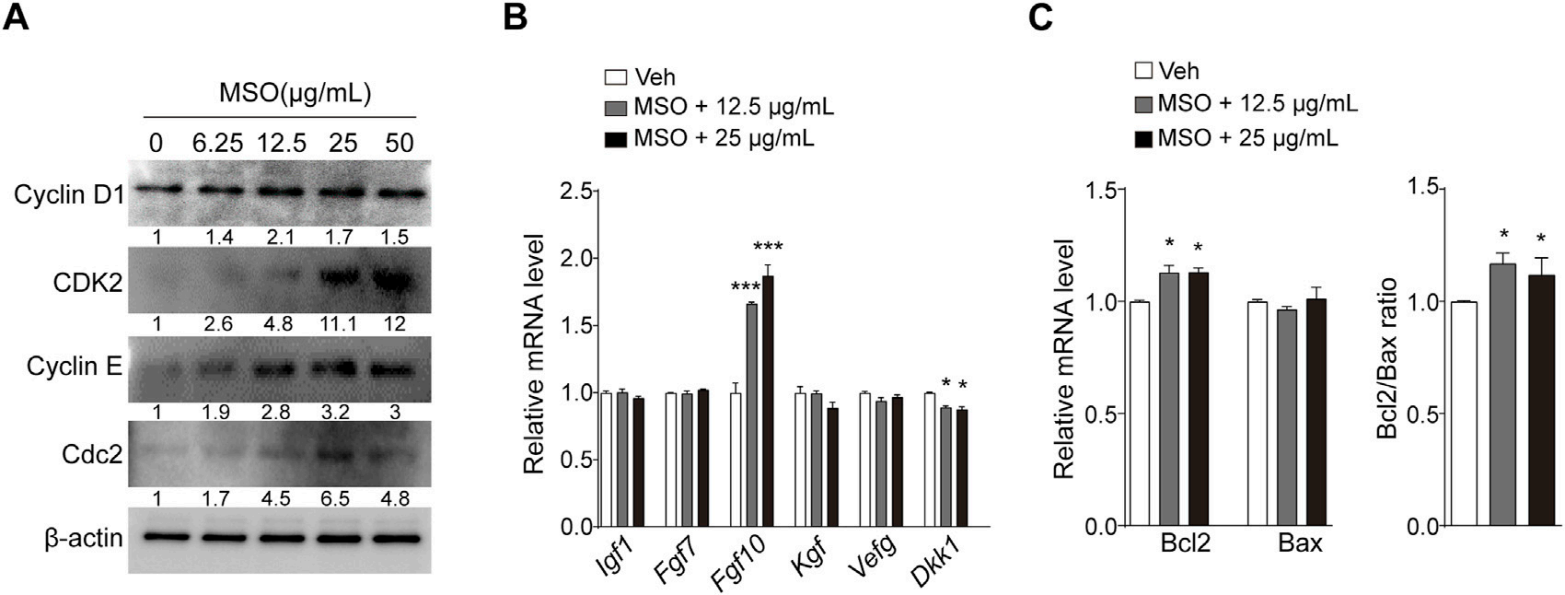
Effect of MSO on growth and apoptosis of HFDPCs. **(A)** Cell cycle-related markers were analyzed by using Western blotting according to MSO concentrations. **(B)** mRNA levels of cell growth factor were analyzed via RT-qPCR. **(C)** mRNA levels of cell apoptosis-related markers were analyzed by using RT-qPCR. Values are represented as mean ± SEM. *, *p* < 0.05; **, *p* < 0.01; ***, *p* < 0.001 compared to the vehicle (Veh) group. Abbreviations: HFDPCs, human hair follicle dermal papilla cells; MSO, millet seed oil.

To investigate the mechanism by which MSO regulates cell growth, the expression of cell cycle-related proteins was measured. Cell-cycle progression is known to be mediated by CDKs and their regulatory cyclin subunits (Malumbres, 2014), and we found that the expression levels of CDK2, cyclin D1, cyclin E, and CDC2 increased in an MSO-concentration-dependent manner (Figure 2A). Next, we investigated whether MSO regulates growth factor genes in HFDPCs. Growth factors mediate the hair-growth cycle and hair morphogenesis (Lin et al., 2015). MSO treatment significantly increased *Fgf10* gene expression at a concentration of 25 μg/mL (Figure 2B). In contrast, the expression of DKK-1 was significantly decreased following MSO treatment. In addition, MSO significantly increased the expression of the anti-apoptotic marker BCL-2 and the BCL-2/BAX ratio (Figure 2C).

### 3.4 MSO promotes hair growth in C57BL/6 mice with dorsal shaved-skin

**FIGURE 3:**
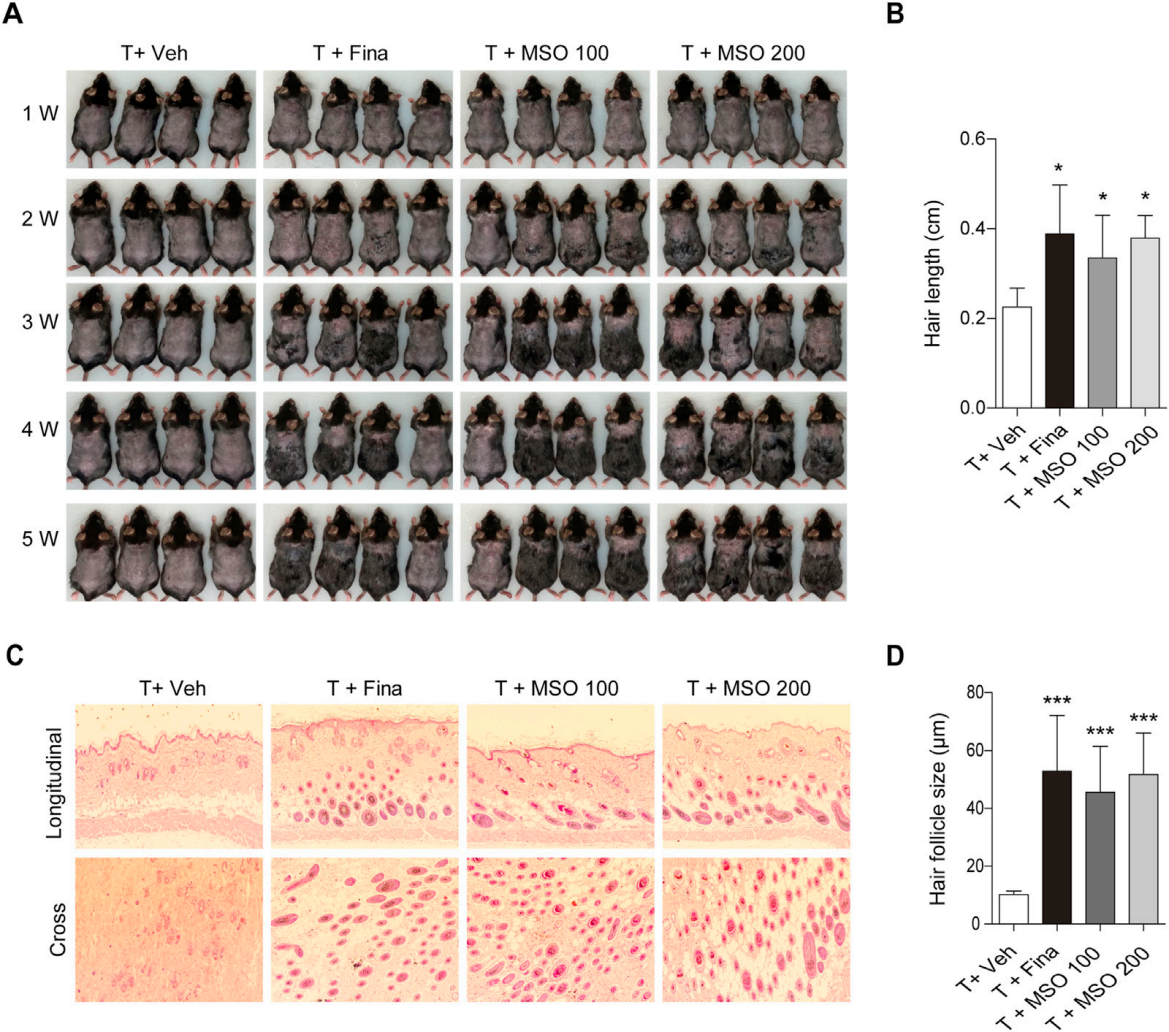
Effect of MSO on hair growth of C57BL/6 mice. **(A)** Hair phenotype after testosterone injection. Photographs were obtained once a week. **(B)** Mouse hair length was measured at 5 weeks. **(C)** The effect of MSO on the hair follicles was analyzed using hematoxylin and eosin (H&E) staining of mouse skin. **(D)** Hair follicle size was measured using CaseViewer software. Values represent mean ± SEM. *, *p* < 0.05; **, *p* < 0.01; ***, *p* < 0.001 compared to the vehicle (T + Veh) group. Abbreviations: MSO, millet seed oil.

We examined the effects of oral administration of MSO on hair growth *in vivo* in the C57BL/6 androgenic alopecia mouse model. The experimental administration of testosterone (T) and MSO did not result in any observable skin disease or tumor morphology in the experimental animals, and measurements of body and liver weight in C57BL/6 mice showed no abnormal changes. Growth of hair shafts was observed from the second week only in the two groups orally administered with MSO (Figure 3A). In the third week, hair did not grow in the vehicle group. In contrast hair growth occurred in the positive control group administered with finasteride. In the vehicle group, hair growth phase transition was inhibited by testosterone administration. Five weeks after depilation, we confirmed that MSO could induce hair growth through the anagen phase transition of hair follicles in mice (Figure 3B). H&E staining indicated that the number of hair follicles in the MSO group was higher than that in the control group (T + Veh) (Figure 3C). Additionally, MSO administration significantly increased the size of hair follicles in the MSO groups compared to that in the control group (T + Veh) (*p* < 0.001) (Figure 3D).

### 3.5 MSO promotes cell cycle progression in mice

**FIGURE 4:**
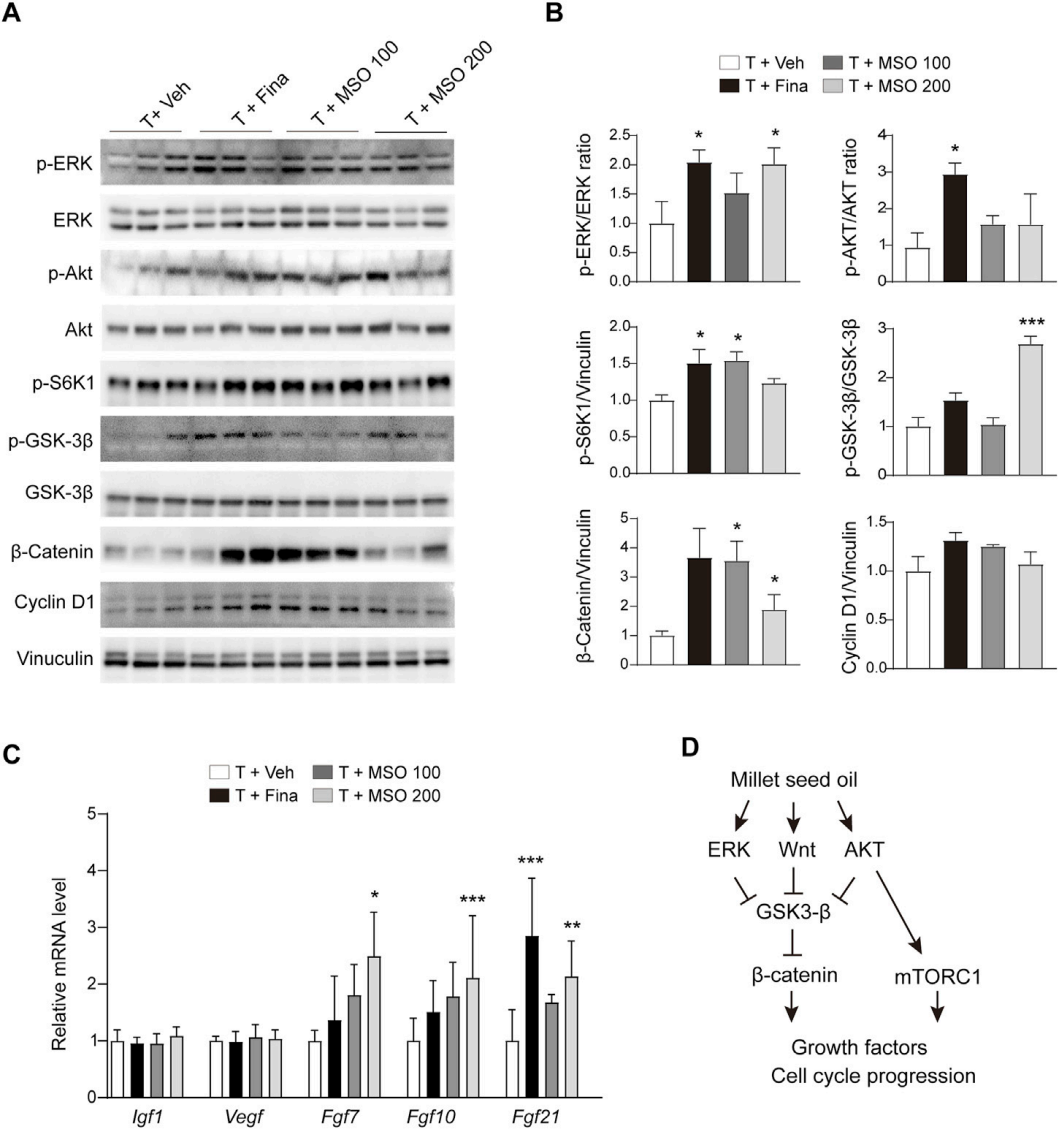
Effect of MSO on hair growth of C57BL/6 mice. **(A)** The Wnt/β-catenin pathway of skin was analyzed using Western blotting. **(B)** Relative protein levels of proliferation-related biomarkers in skin were observed using ImageJ software. Values represent mean ± SEM. *, *p* < 0.05; **, *p* < 0.01; ***, *p* < 0.001 compared to the vehicle (T + Veh) group. Abbreviations: MSO, millet seed oil. **(C)** mRNA levels of growth factor in skin were analyzed via RT-qPCR. **(D)** Schematic overview of this study. MSO activates β-catenin signaling and promotes hair growth.

Total protein was extracted from the back skin of the mice, and protein expression related to cell growth was analyzed by using immunoblotting. MSO showed a pattern of increased phosphorylation of ERK, S6K1, and GSK-3β in skin tissue with significant differences at some concentrations but not at others (Figures 4A,B). AKT phosphorylation was significantly increased in the T + Fina group, but no significant change was observed after MSO treatment. MSO significantly increased β-catenin expression, which was reduced by subcutaneous testosterone injection in mice. We also observed a significant increase in members of the fibroblast growth factor family, such as *Fgf7*, *Fgf10*, and *Fgf21*, in the T + MSO 200 group (Figure 4C). These results suggest that MSO contributes to the activation of cell cycle progression and growth factor expressions.

## 4 Discussion

MSO treatment increased cell proliferation, and the BrdU assay confirmed that MSO treatment induced DNA synthesis in HFDPCs. We propose that the hair-promoting effect of MSO is through the activation of cell cycle progression markers. MSO contains a representative substance called miliacin, a natural triterpenoid known to have healing properties and anti-inflammatory effects (Nuzov, 1990). Through BrdU uptake studies in human keratinocytes, previous studies have shown that miliacin promotes cell proliferation and metabolic capacity (Obrigkeit et al., 2006), suggesting its role in the proliferative effect of MSO in HFDPCs. In addition, miliacin protects cells from apoptosis by lowering DNA fragmentation via blocking the cell death cascade (Panfilova et al., 2003). Apoptosis in hair follicles plays a major role in the process of androgenic hair loss (Kwack et al., 2008). We show that the MSO treatment increases the anti-apoptotic marker BCL-2 in HFDPCs. MSO also appears to induce anagen and hair growth by downregulating the level of DKK-1, an antagonist of the Wnt/β-catenin pathway (Shin et al., 2014).

We also found that hair growth inhibited by testosterone was stimulated by MSO in C57BL/6 mice with shaved dorsal skins. The number and size of hair follicles reduced by testosterone injection was restored by oral administration of MSO. MSO increased the expression of proteins associated with cell growth, which was consistent with the *in vitro* results. Notably, the β-catenin signaling pathway is crucial in hair follicle initiation, development, and growth (Millar et al., 1999; Kishimoto et al., 2000). The induction and duration of anagen, along with the regulation and differentiation of keratinocytes, is promoted by β-catenin expression in the dermal papilla (Bejaoui et al., 2019). MSO appears to promote hair growth by stimulating the β-catenin signaling pathway. Inactivation of β-catenin in the dermal papilla of hair follicles dramatically reduces the proliferation of the hair shaft, resulting in premature induction of the catagen phase (Enshell-Seijffers et al., 2010). In addition, β-catenin activity directly regulates the differentiation of hair follicle stem cells into hair follicle cells (Povelones and Nusse, 2002). It seems that MSO triggers the progression of telogen hair follicles in the mouse skin to the anagen phase, which promotes hair follicle regeneration and growth (Soma et al., 2012; Xiong et al., 2014).

mTORC1 is a key regulator of proliferation, growth, and cell survival and mediates downstream factors such as 4EBP1 and S6K1 (Sarbassov et al., 2005; Kellenberger and Tauchi, 2013). MSO has been shown to increase S6K1 phosphorylation both *in vitro* and *in vivo*, implying that it increases mTORC1 activity. When mTORC1 signaling is activated during the telogen–anagen transition of hair follicle stem cells (HFSCs), they regulate cell activation by balancing BMP-mediated inhibition in regenerating hair (Wang et al., 2022). BMP and WNT/β-catenin signaling mutually regulate differentiation of HFSCs and epidermal regeneration (Wu et al., 2019). The BMP signaling pathway inhibits hair follicle regeneration (Daszczuk et al., 2020). Therefore, MSO may suppress BMP signaling by activating mTORC1 signaling in mice. However, further studies are required to fully understand the mechanisms by which mTORC1 regulates hair growth and the interplay between mTORC1 and other cellular pathways in the context of hair growth.

Activation of the β-catenin pathway increases the expression of genes that mediate cell-cycle progression (Behrens et al., 1996; Behrens and Lustig, 2004), thereby enhancing hair follicle morphogenesis and regeneration. β-catenin forms complexes with GSK3β, CK1, β-Trcp, APC, and Axin in the cytosol. In the absence of Wnt ligands (Wnt OFF state), β-catenin is subsequently phosphorylated by GSK3β and CK1, and phosphorylated β-catenin is recognized by ubiquitin E3 ligase β-Trcp, promoting its ubiquitination and proteasomal degradation (Gao et al., 2014). In the presence of Wnt ligands (Wnt ON state), the ligands bind to Fz and LRP5/6 co-receptors. β-catenin is released from the complex, translocated to the nucleus, and induces the expression of proliferation-related genes (Gao et al., 2014). We confirmed that MSO increased β-catenin expression in HFDPCs under both starvation and nutrient-rich conditions. In particular, the translocation of cytosolic β-catenin to the nucleus was confirmed by immunostaining, suggesting that MSO increases the expression of cell cycle progression-related markers, such as Cyclin D1, CDK2, Cyclin E, and Cdc2. MSO also activates β-catenin signaling related to hair growth in the hair follicles of mice with testosterone-induced androgenetic alopecia, suggesting that MSO can help promote the hair growth cycle and alleviate hair loss. These results indicated that MSO promotes cell-cycle progression by inhibiting β-catenin degradation through the inhibition of GSK3-β by ERK, and AKT, and promoting the nuclear translocation of β-catenin (Figure 4D). Additionally, it also suggests that mTORC1 signaling might also be involved in MSO-induced cell proliferation and growth.

## 5 Conclusion

Taken together, we suggest that MSO promotes hair growth by regulating ERK, AKT, mTORC1, GSK3β, and cell cycle-related markers both *in vitro* and *in vivo*. Notably, MSO was observed to improve hair growth and thickness in mice with hair growth inhibited by testosterone. Our results suggest that MSO can be used as a therapeutic that can help prevent or treat hair loss in alopecia.

## Data Availability

The original contributions presented in the study are included in the article/Supplementary Materials, further inquiries can be directed to the corresponding author.
