# Supplementary figures and images for: Millet seed oil activates β–catenin signaling and promotes hair growth
Source: Front Pharmacol. 2023 May 9;14:1172084. doi: 10.3389/fphar.2023.1172084 (PMC10203242; doi:10.3389/fphar.2023.1172084)

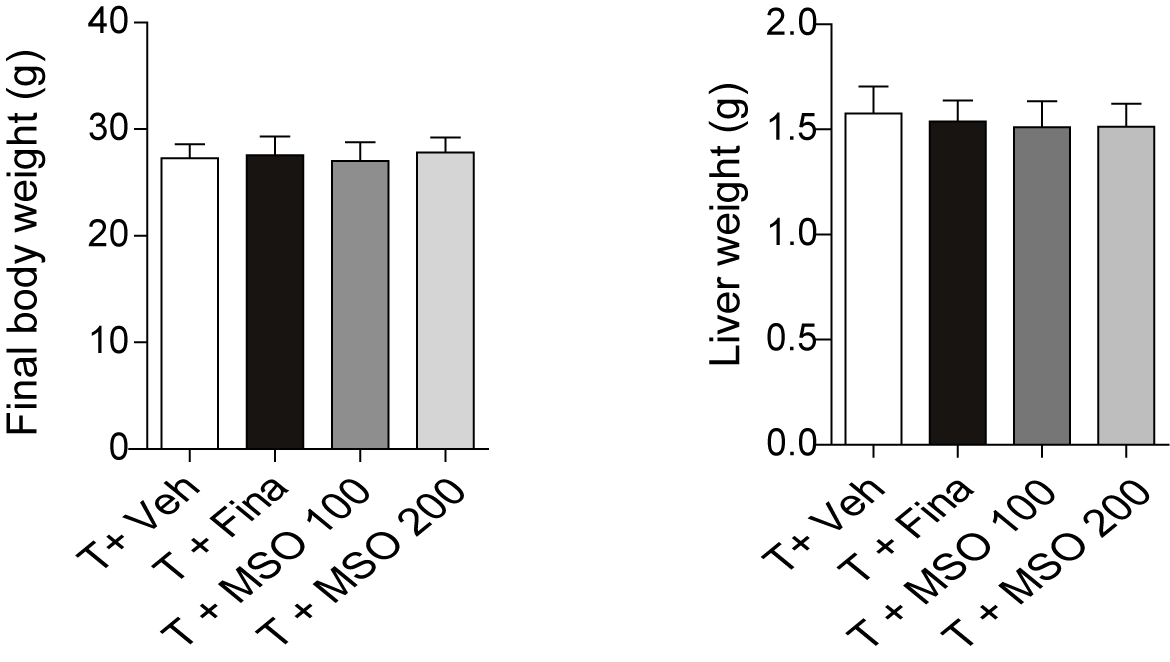

Supplement: Supplementary file 1 [file Image1.TIF]
